# Aortic Aneurysm: Finding the Right Target

**DOI:** 10.3390/biomedicines11051345

**Published:** 2023-05-03

**Authors:** Elena Kaschina

**Affiliations:** 1 Cardiovascular-Metabolic-Renal (CMR)-Research Center, Institute of Pharmacology, Corporate Member of Freie Universität Berlin, Humboldt-Universität zu Berlin, Charité—Universitätsmedizin Berlin, 10115 Berlin, Germany; elena.kaschina@charite.de; Tel.: +49-30-450-525-024; 2 DZHK (German Centre for Cardiovascular Research), Partner Site Berlin,10115 Berlin, Germany

This Special Issue of *Biomedicines* highlights many important scientific findings in aneurysm research. The issue publishes a systematic, up-to-date review on aortic aneurysms (AAs) and nine research articles on pathophysiology, gene expression, novel drug targets, clinical imaging, and prognostic methods. These investigations focus on different segments of the aorta, from the aortic root to the abdominal aorta, as well as on unruptured, ruptured, and repaired aneurysms. The issue also highlights the recent novel methods developed for the evaluation of aneurysm study outcomes. I would like to summarize and briefly discuss the articles in this Special Issue.

Abdominal AAs, being much more common than thoracic aneurysms and dissections [1], have also received a lot of attention in this issue. To begin with, the review by Kessler V. and co-authors [2] provides an appraisal of the literature on risk factors for abdominal AAs, their clinical manifestations, methods of diagnosis, and options for surgical interventions. The authors comprehensively describe the most relevant and up-to-date information on the key pathomechanisms of aneurysm formation, such as intraluminal thrombosis, increased proteolysis, and chronic inflammation, pointing to the role of activated monocytes, macrophages, and neutrophils. The authors also discuss the repurposing of the anti-diabetic drug metformin with anti-inflammatory pleiotropic properties, which may possibly be used for the prevention of aneurysm progression. 

The aortic diameter of abdominal AA is a very important diagnostic parameter, as the risk of rupture increases with its size [1]. Jusko M., with co-authors [3], was looking for the diagnostic criteria, indicating the high risk of rupture. The diameters of the aneurysm and normal aorta were measured by computer tomography and compared in patients with ruptured and unruptured aneurysms. The authors found that in small aneurysms with a present neck segment, the ratio of the aneurysm sac to aorta diameter was a significant prognostic factor for aortic rupture. This work will help surgeons weigh the risk-to-benefit ratio of treating abdominal AA.

A secondary rupture may occur in patients after surgical treatment as well. A perfusion of the aneurysm after endovascular repair (EVAR), an endoleak, often triggers a secondary rupture. The special mechanisms that provoke this added complication and the ways of preventing it are unclear. Buerger M. et al. [4] investigated the structure of aortic tissues in patients after EVAR. The authors evaluated the peptide signature of the thoracic and abdominal aortas by using matrix-assisted laser desorption (MALDI) or ionization mass spectrometry imaging (MSI). Aortas after EVAR were characterized by decreased content of actin, tropomyosin, troponin, and collagen, as well as impaired respiratory chain function. These findings warrant further investigations into possible treatment options for patients with repaired aneurysms. 

Knowledge of genetic architecture may advance understanding of the processes involved in aneurysm formation. Two in silico studies coming from the group of Michael Keese represent the key gene related to abdominal AA. 

First, Li L. and co-authors [5] found the key modules, for example, the mitotic cell cycle, GTPase activity, and several metabolic processes, which may contribute to aneurysmal growth. Furthermore, the authors could identify seven key genes (CCR5, ADCY5, ADCY3, ACACB, LPIN1, ACSL1, and UCP3) that regulate disease progression and 35 compounds targeting these genes. These substances may be candidates for the prevention of aneurysm progression. Kan K.J. et al. [6] further identified significant genes in abdominal AA patients. Moreover, the authors could predict the potential therapeutic compounds for these genes. Weighted correlation network analysis (WGCNA) and text mining identified 3 hub modules and 144 hub genes. The most interesting hub genes were asparagine synthetase, axin-related protein 2, melanoma cell adhesion molecule, and testis-specific Y-encoded-like protein 1. Importantly, potential compounds targeting the genes were also defined: asparaginase, prednisolone, and abiraterone. Indeed, these novel candidates should be further tested in experimental models of aneurysms. 

A genetic study by Piacentini L. and co-authors [7] focused on perivascular adipose tissue that is known to be involved in the pathogenesis of abdominal AA [8]. The authors identified the most relevant transcription factors NFKB1, SPIB, TBP, and the nuclear receptor RXRA, as well as the protein kinases MAPK1 and GSKB3. These factors are known to regulate gene subsets of immune response in the perivascular adipose tissue of abdominal AA. 

The need for the development of effective novel drugs for abdominal AA remains urgent. Here, two studies analyze the effectiveness of new therapeutic approaches.

The first one is pentagalloyl glucose (PGG), a gallotannin, which prevents the degradation of elastin and collagen in blood vessels and restores the biomechanical properties of the arterial extracellular matrix [9]. Recent experimental studies investigated the effectiveness of this compound in abdominal AA. The work by Golledge J. and co-authors [10] performed a meta-analysis of eleven studies on the effects of PGG on abdominal AA expansion. Aortic expansion assessed by direct measurement was used as the primary outcome in this study. The authors additionally analyzed the effects of PGG delivery in specific forms and at different treatment regimes and tested them in different animal models. The authors concluded that the studies were inconsistent. Unfortunately, the evidence that PGG may be protective in patients with early-stage abdominal AA is of low quality. Thus, more information and qualitative research on PGG are needed.

Melin L.G. et al. [11] performed an experimental study in the rat using an elastase perfusion model of abdominal AA. The rats were treated with cycloastragenol, a compound from Astragalus, a Chinese medicinal herb, which improves the functioning of the immune and cardiovascular systems and boosts immunotherapy for some types of cancer [12]. The authors showed that treatment with cycloastragenol decreased aneurysm diameter, matrix metalloprotease-2 activity, reduced calcification, and preserved elastin content in the aorta. In view of these positive effects, cycloastragenol was suggested for a trial in abdominal AA patients.

Two further studies focused mainly on thoracic aneurysms. Parikh S. and co-authors [13] evaluated a new intra-operative video-based method to assess local biaxial strains of the ascending thoracic aorta. The authors performed repeated biaxial strain measurements on the patients undergoing open-chest surgery. Obtained data enable further investigations on the remodeling processes in the thoracic aorta and biomechanical modeling of aortic aneurysms. 

Interestingly, diabetes mellitus is associated with a reduced risk of AA and dissection [14,15]. Therefore, Ntika S. and co-authors [16] were looking for the underlying mechanisms for the reduced risk of thoracic AA in diabetic patients. In aortic tissues from patients with type 2 diabetes, the authors found an increased expression of Syndecan-1 and a marker of macrophages. Syndecans are cell surface proteoglycans that interact with integrins, tyrosine kinase receptors, and extracellular matrix proteins [17]. The authors propose that increased aortic Syndecan-1 expression in humans may reduce the prevalence of thoracic AA in diabetes patients. Given that Syndecan-1 demonstrated protective anti-inflammatory function in aneurysm formation in animals [18], it needs further research as a promising drug candidate.

Altogether, genetic and basic science research presented in this issue identified novel pivotal cell signaling pathways in AAs and new drug targets to prevent aneurysmal growth. Cycloastragenol, Syndecan-1, and pentagalloyl glucose, being promising candidates, require further investigations. Perivascular adipose tissue remains an important source of gene subsets influencing innate and antigen-driven immune responses. Further identification of drug targets that could prevent distal and proximal aneurysmal extension following surgical correction is of great relevance. Moreover, modern scientific approaches described in this issue, such as biomechanical modeling, MALDI-MSI, and weighted correlation network analysis, may pave the way for the development of effective new treatments. 

As the editor, I would like to take this opportunity to express my deep gratitude to all researchers for their great discoveries despite the challenges of COVID-19.
